# The Emerging Role of Extracellular Vesicles and Autophagy Machinery in NASH—Future Horizons in NASH Management

**DOI:** 10.3390/ijms232012185

**Published:** 2022-10-12

**Authors:** Eleni-Myrto Trifylli, Anastasios G. Kriebardis, Evangelos Koustas, Nikolaos Papadopoulos, Melanie Deutsch, Georgios Aloizos, Sotirios P. Fortis, Effie G. Papageorgiou, Ariadne Tsagarakis, Spilios Manolakopoulos

**Affiliations:** 1 Laboratory of Reliability and Quality Control in Laboratory Hematology (HemQcR), Department of Biomedical Sciences, Section of Medical Laboratories, School of Health & Caring Sciences, University of West Attica (UniWA), Ag. Spyridonos Str., 12243 Egaleo, Greece; 2 First Department of Internal Medicine, 417 Army Share Fund Hospital, 11521 Athens, Greece; 3 2nd Department of Internal Medicine, Hippokration General Hospital of Athens, Medical School, National and Kapodistrian University of Athens, Leof. Vasilissis Sofias Avenue Str., 11527 Athens, Greece; 4 Beth Israel Deaconess Medical Center, Harvard Medical School, Boston, MA 02215, USA

**Keywords:** extracellular vesicles, autophagy, non-alcoholic fatty liver disease, non-alcoholic steatohepatitis, liver injury, steatosis

## Abstract

Non-alcoholic fatty liver disease (NAFLD) is considered the most frequent chronic hepatic disease in the general population, while it is the first cause of liver transplantation in the US. NAFLD patients will subsequently develop non-alcoholic steatohepatitis (NASH), which is characterized by aberrant hepatocellular inflammation with or without the presence of fibrosis. The lack of specific biomarkers and therapeutic strategies makes non-alcoholic steatohepatitis (NASH) management a difficult task for clinicians. Extracellular vesicles (EVs) constitute a heterogenic population of vesicles produced by inward or outward plasma-membrane budding. There is an emerging connection between autophagy EVs production, via an unconventional non-degradative procedure. Alterations in the amount of the secreted EVs and the cargo they carry are also involved in the disease progression and development of NASH. Autophagy constitutes a multistep lysosomal degradative pathway that reassures cell homeostasis and survival under stressful conditions, such as oxygen and energy deprivation. It prevents cellular damage by eliminating defected proteins or nοn-functional intracellular organelles. At the same time, it reassures the optimal conditions for the cells via a different mechanism that includes the removal of cargo via the secretion of EVs. Similarly, autophagy machinery is also associated with the pathogenetic mechanism of NAFLD, while it has a significant implication for the progression of the disease and the development of NASH. In this review, we will shed light on the interplay between autophagy and EVs in NASH, the emerging connection of EVs production with the autophagy pathway, and their possible manipulation for developing future therapeutic strategies for NASH.

## 1. Introduction

Non-alcoholic fatty liver disease (NAFLD) comprises a spectrum of heterogeneous hepatic disorders developed due to fat deposition in the liver parenchyma. The variations between the entities of NAFLD emerge from the distinct histopathological aspects of these diseases. Alcoholic fatty liver (NAFL) is characterized by an isolated accumulation of fat, which is termed liver steatosis. Gradually, lipotoxicity induces the injury and inflammation of the hepatocytes, as well as an alteration in their morphology, or so-called “ballooning”. The aforementioned aspect of the disease is called non-alcoholic steatohepatitis (NASH), which can be accompanied by pericellular fibrosis, gradually leading to bridging fibrosis, and finally to cirrhosis development [1]. Additionally, chronic inflammation and fibrosis are closely associated with a higher risk for hepatocellular carcinoma (HCC). The pathogenetic mechanism of NAFLD-related HCC involves the combination of genetic modifications, oxidative stress, and inflammatory and immune responses, as well as autophagy [2]. However, HCC does not necessarily appear only in cirrhotic patients, and constitutes up to 50% of NAFLD-associated HCC cases [3].

Extracellular vesicles are lipid-enveloped nanostructures characterized by a great amount of heterogeneity, including their biogenetic mechanism and their size, as well as the type of cargo that they transfer. Nevertheless, they significantly participate in intercellular communication between the parental cell, which releases them and the recipient cells. This phenomenon is induced by the transferred cargoes, which can either be soluble proteins, lipid molecules, DNA material, autophagosomes, RNA, or messenger or micro-RNAs (non-coding RNAs), as well as long non-coding RNA (lncRNA) [4].

Additionally, autophagy is a multi-stepped lysosomal degradation pathway that facilitates cell survival under extreme conditions, such as nutritional and oxygen deprivation, and reassures the elimination of misfolded or unfolded proteins, and the removal of damaged, non-functional intracytosolic organelles, which could be hazardous for the cell [5]. Meanwhile, a specialized autophagy pathway that is crucial for hepatic lipid homeostasis is so-called lipophagy, which can be down- or upregulated under several conditions that can either have a protective or promoting role for NASH [6].

There is an emerging role between the autophagy pathway and the production of EVs for the secretion of several cargos under stressful conditions for the cell. Under these injurious conditions, such as the accumulation of harmful molecules, the autophagy pathway induces their clearance via the secretion of exosomes, which is a subpopulation of EVs. This autophagy process is considered non-canonical, as it skips the Golgi complex and the endoplasmic reticulum [7].

EVs have a key role in intercellular communication as they transfer cargo between the parental and the recipient cell, while the interaction with the recipient cell can induce or suppress several signaling pathways that modify the cell’s functional state and its phenotypic characteristics. This phenomenon frequently leads to pathogenesis, including NAFLD, while it can also promote the evolution of the disease toward NASH. The physiological EV secretion from hepatocytes assures the survival of hepatocytes; however, aberrations in the amount of produced EVs are closely associated with lipotoxicity. Furthermore, it contributes to the enhancement of the inflammatory state and the fibrotic injury of the hepatic parenchyma, which leads to disease progression [8].

In this review, we will shed light on the interplay between EVs and the macroautophagy machinery, and their contribution to NASH progression, as well as the therapeutic opportunities that open up via their manipulation in NASH management.

## 2. An Overview of the Conventional Macroautophagy and Lipophagy Pathway

Autophagy constitutes a lysosomal degradation pathway, which includes multiple steps with a great extent of regulation, which serves as a crucial homeostatic mechanism that assures cell survival under stressful conditions such as inflammation, oxygen deprivation, and nutrient deficiency. At the same time, it also degrades potentially harmful misfolded proteins or defective organelles [9].

**Figure 1:**
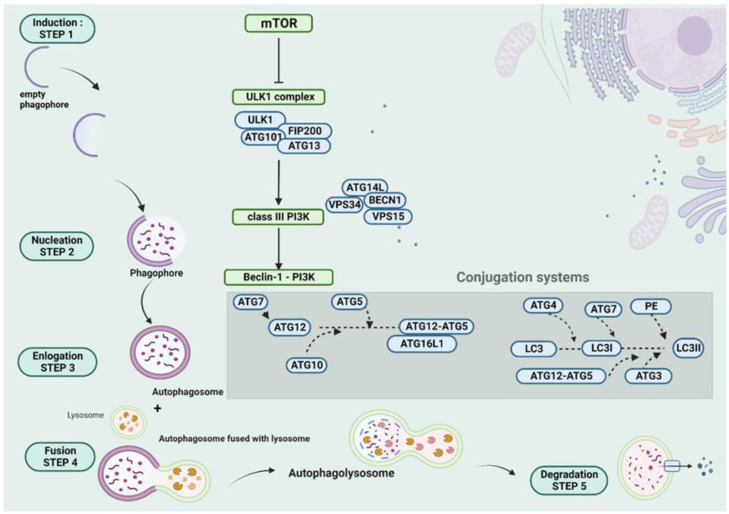
A schematic presentation of the autophagy steps. The autophagy pathway is inducted via the inhibition of mTOR, which is dissociated from ULK1. The second step of nucleation of ULK1 activates the class III PI3K, with the formation of the Beclin-1-PI3K complex. The third step includes the elongation of the phagophore and the formation of autophagosome. Two conjugations take place at that level, between ATG12 and ATG5, as well as LC3I and PE. The fourth and the fifth step include the fusion of lysosome with autophagosome, resulting in an autophagolysosome and the degradation of cargoes, respectively. This figure was created with “BioRender.com” accessed on 24 August 2022. (Agreement number OR24BL4UPR).

The macroautophagy pathway starts with the induction step, which includes the inhibition of the mammalian target of rapamycin (mTOR) and its dissociation from ULK1, which is part of the ULK1 complex (ULK1, ATG13, ATG101, and FIP200). After the dissociation of mTOR from ULK1, the latter is active and it further phosphorylates the class III PI3K complex (BECN1, VPS15, and VPS34, and ATG14L), followed by the formation of the Beclin-1-PI3K complex. The aforementioned step is called nucleation, and it includes the recruitment of the cargo by the previously empty phagophore to the so-called phagophore assembly site (PAS). The elongation of the phagophore membrane constitutes the third step, in which two conjugations occur between ATG8 (microtubule-associated protein 1 light chain 3 (LC3)) and PE, as well as between ATG12 and ATG5. The autophagosome is formed and matured at that level, which is achieved via the two conjugation systems. After the activation of ATG12 by ATG7, which interacts with a ubiquitin-like conjugating enzyme E2 (ATG10), ATG5 is covalently conjugated (irreversibly) with ATG12. Afterward, ATG16 forms a complex with the conjugated ATG12-ATG5. Compared to the aforementioned conjugation system, LC3I is reversibly conjugated with PE, while ATG4 facilitates deconjugation. Firstly, a cleavage of LC3 by ATG4 is required, which forms LC3I. ATG7 also achieves the activation of LC3I, and the role of ubiquitin-like conjugating enzyme E2 has ATG3. The participation of ATG12–ATG5 and ATG3 facilitate the conjugation of LC3I with PE. After the formation of the autophagosome and the cargo recruitment, there is a fourth crucial step in the formation of autophagolysosome, which occurs after the union of lysosome with autophagosome. The ultimate step is the degradation of autophagolysosome’s cargo and the release of the degradation products in the extracellular compartment [10,11,12]. An illustration of autophagy machinery is demonstrated in Figure 1.

**Figure 2:**
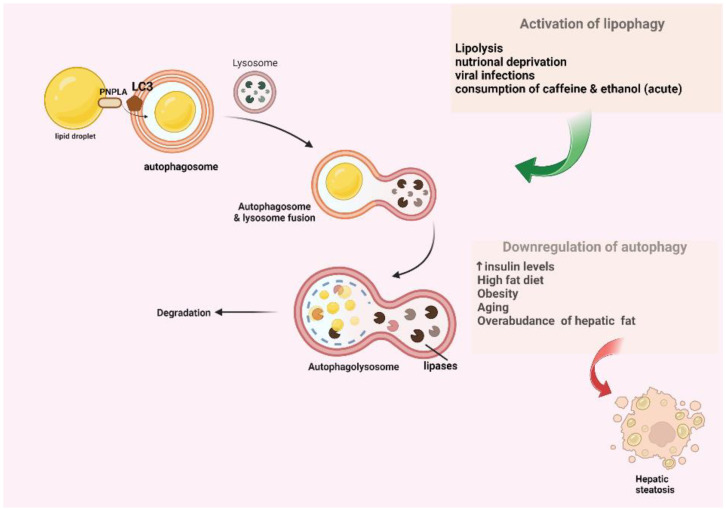
A schematic presentation of lipophagy. Autophagy pathway is closely related to NAFLD. Autophagy is downregulated in circumstances that favor NASH, such as increased insulin levels, obesity, increased consumption of lipids, as well as aging and overabundance of lipid storage in the liver parenchyma. The LDS are recognized and engulfed into autophagosomes via the interaction between LC3 and PNPLA. Then, fusion occurs between autophagosome and lysosome, resulting in autophagolysosome, which subsequently leads to the degradation of LDs by lipases. This figure was created with “BioRender.com”, accessed on 29 September 2022. (Agreement number ZS24GPTKTR).

Lipophagy is another specialized homeostatic mechanism that is crucial for lipid droplets (LDs) that are located not only in the adipocytes, but also in the hepatocytes. The LDS are recognized and targeted as cargo via interactions with the LC3, which is located on the membrane of autophagy. These LDs are engulfed in autophagosomes, which occur via the interaction between LC3 and the PNPLA motif on the surface of LDs. Afterward, a fusion occurs between autophagosome and lysosome, resulting in autophagolysosome formation, and subsequently, in the degradation of LDs by the lysosomal enzymes [6,13,14]. There is a schematic presentation of the lipophagy pathway demonstrated in Figure 2.

However, lipid droplet homeostasis is also achieved via the recognition of apolipoprotein-B100 as cargo and its degradation [15]. Moreover, it was demonstrated in mice that the knockout of transcription factor EB (TFEB) that regulates Atg7 and Atg14 leads to the suppression of autophagy and the disruption of lipid homeostasis in the liver, resulting in the accumulation of cholesterol and triglycerides, as well as in the development of steatosis, and also in alcohol-related cases. However, the reversion of steatosis was observed after the upregulation of these autophagy-related proteins [16,17]. However, there are some studies that did not prove that autophagy suppression leads to steatosis, a phenomenon that was attributed to several characteristics of the animal models [18].

## 3. The Association between Autophagy and NASH

Based on various studies on the impact of the autophagy pathway on NAFLD, it is demonstrated that autophagy is closely associated with NASH progression via several factors that either promote or inhibit the autophagy pathway. The downregulation of autophagy is observed in many circumstances that favor NASH, such as increased insulin levels [19], obesity [20], the increased consumption of lipids [20,21], as well as aging [22] and the overabundance of stored lipids in the liver. The downregulation of the pathway is estimated via the assessment of LC3II and autophagosomes, as well as the number of autophagolysosomes. The reduction in autophagy in fatty liver is possibly attributed to the compromised formation of autophagolysosomes resulting from an impaired integration of the lysosome with the autophagosome, the decreased amount of lysosomal degradative enzymes, and the downregulation of the expression levels of autophagy-related genes. Moreover, it is demonstrated in animal models (murines) with NAFLD that the suppression of the autophagy pathway in hepatocytes noticeably leads to the aggravation of steatosis. On the contrary, the activation of the pathway promotes hepatic lipid metabolism. Some of the circumstances that promote the hepatic autophagy pathway include: conditions of lipolysis and nutritional deprivation, and viral infections, as well as the consumption of caffeine and acute ethanol intake [18,23]. The possible protective role of chronic hepatitis B infection should be underlined, implied by the low incidence of steatosis, which is attributed to metabolic profile modifications. However, hepatitis C virus (HCV) infection alters lipid metabolism and promotes insulin resistance, which further downregulates autophagy and induces steatosis progression [24]. Further research is needed for shedding light on the linkage between HBV–autophagy modulation and NASH progression.

## 4. An Overview of Extracellular Vesicle Biogenetic Mechanisms

EVs constitute multi-sized nanoparticles that are characterized by a great amount of heterogeneity, including the manner of their biogenesis, their size, and their cargo, while they are secreted by several cell types. They significantly participate in intracellular communication between the parental and recipient cells, which is achieved through the transferred cargo. The aforementioned cargo can be either genetic material, including DNA and RNA molecules, mitochondrial DNAs (mtDNAs), micro-RNAs (non-coding RNAs), long non-coding RNA (lncRNA) or autophagosomes, proteins, and lipid molecules [25]. Based on their biogenetic mechanisms and sizes, they are sub-classified into three distinct entities: (i) the apoptotic bodies, with a size over 1000 nm, (ii) the microvesicles that are sized between 150 and 1000 nm, as well as (iii) the exosomes, which are sized between 40 and 150 nm. The mechanism of biogenesis is different for each subclass of EVs, resulting from inward membrane budding for exosomes, outward budding for the microvesicles, and a cell apoptotic mechanism for the apoptotic bodies [26].

More particularly, exosome formation begins with the inward budding of the membrane via the contribution of the endosomal network. This endocytic pathway includes the internalization of the specific transmembrane proteins into microvesicles, which are separated from the cell membrane and integrated with the early endosomes. The early endosome defines the providence of these internalized molecules, which are either delivered back to the cell membrane or further directed into the intraluminal vesicles (ILVs) [27]. The following steps include the formation of ILVs and multivesicular bodies (MVBs) which require the involvement of an endosomal sorting complex (ESCRT) complex that is composed of ESCRT 0, I–III. The aforementioned complex have a key role in membrane remodeling and MVB generation. ILV formation requires the involvement of ESCRT-0, which further recruits ESCRT-I, and the contribution of ESCRT-II for the invagination of the endosomal membrane. Additionally, MVB formation requires the recruitment of ESCRT-III by ESCRT-II, which also participates in the ILVs splitting into the lumen of MVE (multivesicular endosome) or MVB, with MVB being a round entity composed of several ILVs [28]. However, there is also an ESCRT-independent mechanism of exosome production, when the cargo is recruited from different sites, such as the Golgi apparatus (trans-Golgi network), the cytoplasm, and the membrane. Finally, MVBs are integrated in the cell membrane, a phenomenon that leads to the secretion of exosomes in the extracellular space via exocytosis. This step requires the involvement of soluble NSF attachment protein receptor (SNARE) proteins such as Ykt6, VAMP7, and syntaxin 1A (Syx1A), which have a key role in the fusion of MVBs with the cell membrane [29,30]. However, MVBs can be possibly led to lysosomes for degradation, or can be fused with autophagosomes [31]. The interconnection between autophagy and EV secretion will be further analyzed later in this review.

Microvesicles are medium-sized EVs that have a different biogenetic mechanism compared to exosomes, starting with the outward budding of the plasma membrane. The outward budding of the plasma membrane is often mediated by the interaction between the arrestin domain-containing protein-1 (ARRDC1) protein and TSG101. This interaction induces the relocation of TSG101 from endosomal membranes to cell membrane, a phenomenon that induces the blebbing of the plasma membrane and the release of microvesicles, which encompass either ARRDC1 or TSG101 proteins. The key regulator of selective cargo trafficking for microvesicles is the ADP-ribosylation factor 6 (ARF6) protein. The main selective recruited cargoes are either nucleic acids or proteins, including major histocompatibility complex (MHC) proteins, and VAMP3, as well as integrin beta-1. Some other proteins that are involved in the induction of blebbing are SNAREs, as well as Rab-GTPases, which are involved in protein recruitment and the release of microvesicles under conditions of oxygen deprivation [31,32,33,34,35].

Meanwhile, cell apoptosis gives rise to a different entity of EVs, which have the largest size, the so-called apoptotic vesicles (ApoEVs) and their sub-portion: the so-called apoptotic bodies (ApoBDs). Cell apoptosis includes the shrinkage and condensation of the chromatin, and the blebbing of the cell membrane, as well as the collapse of the nucleus and the disintegration of the cell organelles. Apoptotic cell disassembly is a multiplex, strictly regulated multi-stepped procedure that includes the blebbing of the cell membrane and the formation of protrusions (beaded apoptopodia, apoptopodia, and microtubule spikes) that lead to the formation of the apoptotic cell body (over 5 μm), which is further fragmented, forming the apoptotic bodies (1–5 μm) [36,37].

**Figure 3:**
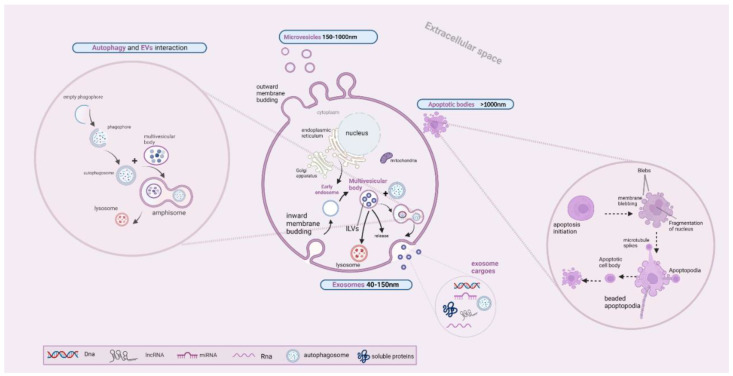
A schematic presentation of EVs biogenetic mechanism. Exosome formation begins with the inward budding of the membrane, with the formation of microvesicles and the internalization of transmembrane proteins. Microvesicles are separated from cell membrane and subsequently form early endosomes with the internalized molecules being either delivered back to the cell membrane or further directed into the intraluminal vesicles (ILVs). The ILVs are split into the lumen of MVB, with MVB being either fused with the cell membrane for the exocytosis of exosomes or being fused with autophagosome and lysosome, forming amphisome, or being led to the lysosome for degradation. Microvesicles biogenesis starts with the outward budding of the plasma membrane, while the production of apoptotic bodies is through cell apoptosis with the formation of the apoptotic cell body and apoptotic bodies. This figure was created with “BioRender.com” accessed on 27 August 2022. (Agreement number QX24C1405A).

Furthermore, EVs significantly contribute to intercellular communication between recipient and parental cells. EVs interact with recipient cells in various manners, such as via ligand–receptor interaction that activates signaling pathways inside the recipient cell, via endocytosis by which EV-contained cargoes are incorporated into the intracellular space, or via other endocytic mechanisms such as micropinocytosis, receptor-associated endocytosis (RME), phagocytosis, and lipid or caveolin-mediated endocytic mechanisms [38]. Although the mechanism of EVs delivery was widely studied, the mechanism by which EVs are incorporated into the cells is still unclear. A better knowledge of the interaction between parental and recipient cells via the EV-contained cargo can give us answers about the mechanisms of disease progression, such as in NAFLD. We demonstrate in Figure 3 the schematic presentation of the EV biogenetic pathways.

## 5. The Interplay between EV Secretion and the Autophagy Pathway

The relationship between the autophagy pathway and autophagy-related proteins was firstly demonstrated in endothelial cells, under the conditions of nutritional deprivation, with a subsequent release of EVs that expressed autophagy markers. It is proposed that cell homeostasis is ensured via the so-called secretory autophagy pathway under specific conditions. The secretory autophagy pathway is related to the production and recruitment of the cargo, and the secretion of EVs [39,40]. Conditions that disrupt and that suppress the endolysosome system, including the fusion between autophagosome and lysosome, or the impaired maturation level of the autophagosome, can lead to the activation of the aforementioned pathway and the secretion of EVs that contain autophagic cargo receptors, such as the p62 protein, which could be otherwise accumulated in the cytoplasm [41,42]. The regulation of the aforementioned pathway is mediated via multiple autophagy-related proteins and Rab27 at the level of autophagosome formation/maturation and the EV exocytosis, respectively [39,40,41,42,43].

More particularly, the interplay between these two pathways is prominent during the biogenesis of exosomes, when the MVB is fused with the autophagosome, forming the amphisome and being further fused with lysosomes. Several autophagy-related proteins have a key role in exosome generation, such as ATG5 and ATG16L1, which are closely associated with the MVBs secretory pathway, inducing the fusion of MVBs with cell membrane and the release of exosomes [44]. It has been also demonstrated that the LC3 autophagy-related protein is involved in LC3-dependent EV loading and secretion (LDELS). The role of the aforementioned protein is illustrated by the fact that is located in the membranes of MVBs, having a regulatory role for the degradation of the endocytosed molecules. Additionally, it also regulates which RNA and protein molecules will be loaded into ILVs for their secretion into the extracellular milieu [45]. The RNA profiling of EVs, as well as proteomic analysis, demonstrated that LC3II (a lipidated form of LC3) is presented in a wide portion of EVs, which implies the significance of LC3II for cargo loading [46,47]. It has to be underlined that LDELS does not require the contribution of any other autophagy-related protein, except for the LC3 conjugation system [46,47,48]. We demonstrate a schematic presentation of autophagy-induced EVs secretion in Figure 3, including the formation of an amphisome via the fusion of the autophagosome and MVB, which is regulated by various proteins such as Rab GTPases, soluble N-ethylmaleimide-sensitive factor attachment proteins (SNAP) receptors (SNAREs), and ESCRTs [49].

## 6. The Association between EVs and NASH

Hepatic steatosis is “globally” manifested (25% of the population)**,** while it is considered as the most commonly diagnosed chronic liver disease nowadays. However, it is still unclear why only a portion of patients with NAFL (30%) develop inflammation of the hepatic parenchyma, with or without fibrotic injury [50,51]. Lipotoxic hepatocytes are considered the major source of EVs production in the circulation. EVs have a crucial role in the disease progression via enhancing the inflammatory state, which is manifested in the liver parenchyma, via inducing monocyte/macrophage chemotaxis, which leads to pro-inflammatory cytokine release (IL-6, IL1-b), as well as via the activation of LSECs and HSCs that promote angiogenesis and fibrotic injury in the parenchyma, respectively. Hepatocytes release EVs under conditions of lipotoxicity, such as in cases of lipid overabundance and excessive storage in the hepatic parenchyma. Lipotoxicity is induced when a wide variety of lipids are stored in the hepatic parenchyma, such as free cholesterol, ceramides, and FAs, as well as diacylglycerols and phospholipids. It is demonstrated that the transferred cargoes possibly interact in different manners with the recipient cells, depending on the stage of the disease. EVs significantly contribute to the activation of various pathogenetic mechanisms and lead to disease progression. Their emerging role in NAFLD and their progression towards NASH has been in the spotlight in recent years [52,53,54,55].

Lipotoxicity induces the disruption of the functional state of hepatocytes and the LSECs, leading to the secretion of EVs that activate the HSCs. The activation of HSCs can be induced via the hepatocyte-derived EVs that contain miR-192 and miR-128-3p, or via sphingosine-1 phosphate (S1P) and sphingosine kinase 1 (SphK1) that are contained in EVs derived from LSECs [56,57,58,59,60]. The activation of HSCs contributes to the progression of NASH and the development of fibrotic injury in the hepatic parenchyma, while it is also induced via the production of HSCs-derived EVs containing fibrogenic proteins. Similarly, some of the cargoes contained in hepatocyte-derived EVs, such as vanin-1 (VNN1), as well as miR-1, also promote fibrogenesis and angiogenesis in the liver parenchyma via activation of the LSECs. Another phenomenon that promotes angiogenesis is the release of VEGF-containing EVs from portal fibroblasts [61,62].

Furthermore, the progression of NASH is also induced by the enhancement of the inflammatory state in the parenchyma, which was demonstrated in animal models. It has been demonstrated that the injection of EVs from mice that had an increased amount of fat in their diet into mice that had a chow diet, enhances inflammation [63]. This aggravation is mainly attributed to the chemotaxis of immune cells induced by the C-X-C motif chemokine ligand 10 (CXCL10) that is contained in hepatocyte-derived EVs. Additionally, there is an association between CXCL10 and Mixed lineage kinase 3 (MLK3) in the pathogenetic mechanism of NASH. More particularly, the former favors the release of CXCL10-contained EVs from the hepatocytes under lipotoxic stress. CXCL10-contained EVs induce macrophage-related inflammation, while the suppression of MLK3 limits their release, and subsequently, the chemotaxis of macrophages [64,65]. In addition, macrophage–related inflammation is also induced via EVs that contain mitochondrial DNA (mtDNA) and miR-192-5p, as well as ceramides, integrin-beta 1, TNF-related apoptosis-inducing ligand (TRAIL), and oxidized mtDNA. The amount of integrin-beta 1-containing EVs is found to be elevated in NASH patients with mild fibrotic injury, compared to patients with steatosis, which is attributed to their association with monocytes recruitment. It has to be noted that the aforementioned phenomenon implies their possible use as disease biomarkers [66,67,68,69]. Moreover, the enhancement of chemotaxis and the aggravation of the inflammatory state is also induced by the transmembrane protein IRE1a, which induces the release of a great amount of EVs in the extracellular milieu. It has to be underlined that IRE1a, as well as MLK3, ROCK1, JNK, and ASK1 constitute NASH-promoting kinases, which are considered as mediators for NASH progression [70,71]. Furthermore, TRAIL-contained EVs induce the activation of macrophages and the NF-κB pathway, while EVs that carry ceramide are similarly involved in macrophage chemotaxis [72,73,74].

Aberrations in EVs amount are closely associated with NAFLD pathogenetic mechanisms, as well as with disease progression. It is observed that NAFLD patients with severe fibrotic injury (grade 3 or 4) have a decreased amount of EVs, which are derived from endothelial cells or leucocytes, compared to NAFLD patients with mild or without fibrotic injury (grade 0/1–2) [75]. There are many studies that demonstrate an aberrant amount of EVs in the circulation of cirrhotic or pre-cirrhotic NASH patients, in comparison with healthy donors. The severity of the disease could be identified either by the number of EVs or via the protein cargo contained in EVs. The aforementioned observations open up new horizons for the utilization of these vesicles as a liquid biopsy for the estimation of NASH advancement and prognosis. Based on the above, EVs could be used as diagnostic and prognostic tools for NASH/NAFLD, while they can be also used for the differentiation of a specific disease among chronic liver diseases [76,77,78].

Furthermore, several cargoes contained in EVs are closely associated with different levels of NAFLD severity, such as miR-128-3p and miR-122, as well as miR-192. More particularly, it was identified that patients with NAFL exhibit less miRNA expression, in comparison with NASH patients, whereas the former present a higher expression than healthy controls. However, the aforementioned miRNAs lack specificity due to the fact that they are not only expressed in hepatic tissue. Between these three miRNAs, miR-122 has the highest specificity due to the fact that it is mainly originated by liver (70% of its total level of expression). MiR-22 levels have been found to be proportionally elevated with disease progression and severity. Similarly, miR-128-3p, miR-16, and miR-34A, as well as miR-192-5p levels, have been also found to be noticeably elevated in patients with NAFL or NASH, in comparison with healthy donors. Meanwhile, an elevated amount of miR-122 and miR-129 levels have been closely related with disease progression and a more advanced stage of disease [79,80]. Likewise, higher expression levels of SLC27A5 and ASGPR1-containing EVs have been linked with disease progression, with the former being a prognostic marker for HCC development and the latter being associated with a significant fibrotic injury [81,82].

Last but not least, it must be underlined that NASH progression can be also induced via the interconnection between lipotoxic hepatocytes-derived EVs with adipose tissue. More specifically, based on in vitro studies, miR-let-7e-5p-containing EVs modify the amount of lipid deposition in preadipocytes, enhance lipogenesis in the adipose tissue, and further lead to disease progression [83]. We present a summary of the effects of EVs on NASH progression in Table 1.

## 7. Future Horizons in NASH Management

As was previously mentioned, EVs have a crucial role in intercellular communication, and can potentially alter the functional states of various cells via their cargoes. Based on the fact that they can transfer a wide variety of cargoes and that they are nanosized particles, as well as their increased biocompatibility and limited immunogenicity, they are considered ideal drug delivery vectors [84]. The manipulation of EVs as drug delivery tools is in the spotlight nowadays, especially for cases of NASH where there is no standardized treatment plan. More particularly, the manipulation of pluripotent stem cells (iPSCs)-derived EVs that contain miRNA cargoes such as miR-302-3p and miR-92-3p induces a notable suppression of fibrogenesis via their endocytosis by HSCs [85]. Another significant example of the utilization of EVs as vectors is the manipulation of MSC-derived extracellular vesicles (MSC-EVs) that can potentially modify the immune responses and limit the inflammatory state in various hepatic diseases, including NASH and autoimmune hepatitis. Their effect is attributed to their suppressive effect on macrophages of M1 phenotype and on pro-inflammatory cytokine release. Moreover, they can induce the multiplication and activation of T-regulatory cells and macrophages of M2 phenotype, limiting the activation of HSCs and Kupffer cells, implying their therapeutic potential in NASH [86,87,88].

Another potential therapeutic strategy is combating the EV-containing cargoes that induce NASH progression, either via the promotion of macrophage chemotaxis that aggravates the inflammatory state, or via fibrosis and angiogenesis promotion. More particularly, potential strategies for combating macrophage chemotaxis, which is induced by CXCL10-contained EVs, includes the utilization of neutralizing CXCL10-antisera or the inhibition of the NASH-promoting kinases such as Mixed lineage kinase 3 (MLK3) that favors their release [64,65,89].

Additionally, the manipulation of fat-laden hepatocyte-derived EVs constitutes another novel therapeutic strategy for fibrosis management in NASH via the targeting of peroxisome proliferator-activated receptor (PRAR) γ, and via the overregulation of the expression of profibrogenic genes [90]. Based on the role of PRAR (α, γ, δ) in lipid and glucose metabolism, targeting PRARs constitutes a promising therapeutic strategy for NASH management [91]. It is demonstrated that PRAR-mesenchymal stem cell-derived extracellular vesicles (iMSC-EVs) significantly improve lipid deposition and steatosis, as well as inflammation via p65 suppression [87]. A promising effect is also demonstrated in a recent study that examines the utilization of human liver stem cells (HLSCs) that can potentially attenuate the hepatic inflammation and parenchymal fibrotic injury in murines [92]. Based on the aforementioned study, mice with NASH demonstrated a notable downregulation of pro-fibrotic genes. Similarly, a reduction in inflammation was also demonstrated via amnion-derived MSCs (AmMSC-EV), which limited the fibrotic injury and the progression of NASH in rats [93].

Moreover, another potential treatment strategy for NASH is mediated through the manipulation of autophagy pathway. The utilization of mTOR inhibitors such as MSDC-0602K (NCT02784444, phase 2 clinical trial) was accessed in the EMMINENCE trial. The aforementioned trial demonstrated the efficacy and safety of MSDC-0602K use in patients with grade 1–3 NASH. However, it did not show any statistically important effects on hepatic histology [94]. Curcumin and YAP-001 were also accessed in NCT04109742 and NCT03962608 in NASH patients, respectively [95,96]. In addition, S-adenosyl-L-methionine utilization was also accessed in a phase 3 clinical trial NCT01754714 in NASH patients, which is closely associated with the inactivation of autophagy [97]. It has to be underlined that the suppression of autophagy is considered harmful for NAFLD, whereas the activation of autophagy via pharmacological and non-pharmacological agents demonstrated favorable effects. Examples of autophagy induction include: autophagy gene modulation via an adenovirus vector, which showed a noticeable decrease in steatosis in animal models [98,99].

Last but not least, there are several ongoing trials for novel autophagy-targeting agents for NAFLD management, such as AMPK activators, including oltipraz, monascin, berberin, and ankaflavin, as well as, quercetin and curcumin. AMPK downregulation is found to be closely related to NAFLD, which was demonstrated in mice with NASH who were given diets with decreased AMPK activity, and presented a progression of liver fibrosis and apoptosis [98,100].

## 8. Conclusions

Hepatic steatosis is manifested in 25% of the global population, and it is considered as the most commonly diagnosed chronic liver disease, nowadays. Lipotoxic hepatocytes are considered as major contributors for EVs release in the blood circulation, which subsequently promotes disease progression via the enhancement of the inflammatory state in the liver parenchyma, via the induction of monocyte/macrophage chemotaxis, which leads to pro-inflammatory cytokine release, as well as via the activation of LSECs and HSCs that promote angiogenesis and fibrotic injury in the parenchyma. Several cargoes contained in EVs can potentially induce the aggravation of inflammation, fibrogenesis, and angiogenesis, implying their significant association with the severity of the disease. The autophagy pathway is also closely related to NAFLD, which is downregulated in circumstances that favor NASH, whereas it normally promotes hepatic lipid metabolism. The manipulation of EVs as drug delivery vectors and the exploitation of autophagy via autophagy modulation, as well as the use of autophagy interconnection with EVs secretion, constitute promising therapeutic strategies for NASH management. However, further research is considered vital for this frequently diagnosed disease, which subsequently leads to cirrhosis and HCC development.

## Figures and Tables

**Table 1:** A summary of the effects of EVs on NASH progression.

Parental Cell	EV-Contained Cargo	Recipient Cell	Effect
Lipotoxic hepatocyte	miR-192 miR-128-3p VNN-1	HSCs	-Activation of HSCs -Progression of NASH and fibrotic injury [56,57,58,59,60]
	miR-1 VNN-1	LSECs	-Activation of LSECs -Promotion of angiogenesis and fibrinogenesis [61,62]
	CXCL10 Ceramides [48] Integrin beta-1 TRAIL Oxidized mtDNA mtDNA miR-192-5p	Macrophages Monocytes	-Chemotaxis -Disease Progression [64,65,66,67,68,69]
	miRNA let-7e-5p	Preadipocytes	↑Lipogenesis [83]
LSECs	S1P [48] SphK1	HSCs	↑Adipocyte Lipogenesis [61,62]
Portal fibroblasts	VEGF	LSECs	-Activation of HSCs -Progression of fibrotic injury and angiogenesis [61,62]

Hepatocyte stellate cells (HSCs), liver sinusoidal endothelial cells (LSECs), vanin-1 (VNN-1), sphingosine 1-phosphate (S1P), Sphingosine Kinase 1 (SphK1), Vascular endothelial growth factor (VEGF), Mitochondrial DNA (mtDNA), C-X-C motif chemokine ligand 10 (CXCL10), TNF-related apoptosis-inducing ligand (TRAIL).

## Data Availability

Not applicable.

## Abbreviations

Amnion-derived MSCs	AmMSC
Apoptotic bodies	ApoBDs
Apoptotic vesicles	ApoEVs
Arrestin domain-containing protein-1	ARRDC1
C-X-C motif chemokine ligand 10	CXCL10
Endosomal sorting complex	ESCRT
Enhanced Liver Fibrosis	ELF
Extracellular vesicles	EVs
Hepatocellular carcinoma	HCC
Human liver stem cells	HLSCs
Intraluminal vesicles	ILVs
LC3-dependent EV loading and secretion	LDELS
Long non-coding RNA	lncRNA
Major histocompatibility complex	MHC
Mammalian target of rapamycin	mTOR
Microtubule-associated protein 1 light chain 3	LC3
Mitochondrial DNA	mtDNA
Mixed lineage kinase 3	MLK3
MSC-derived extracellular vesicles	MSC-EVs
Multivesicular bodies	MVBs
Multivesicular endosome	MVE
Non-alcoholic fatty liver	NAFL
Non-alcoholic fatty liver disease	NAFLD
Non-alcoholic steatohepatitis	NASH
Peroxisome proliferator-activated receptor	PRAR
Phagophore assembly site	PAS
Pluripotent stem cells	iPSC
Soluble NSF attachment protein receptor	SNARE
Sphingosine kinase 1	SphK1
Sphingosine-1 phosphate	S1P
Transcription factor EB	TFEB
Trans-Golgi network	TGN
Tumor susceptibility gene 101 protein	TSG101
Vanin-1	VNN1
